# Supplementary material for: Efficacy and safety of palbociclib and ribociclib in patients with estrogen and/or progesterone receptor positive, HER2 receptor negative metastatic breast cancer in routine clinical practice
Source: PLoS One. 2021 Jul 22;16(7):e0253722. doi: 10.1371/journal.pone.0253722 (PMC8297817; doi:10.1371/journal.pone.0253722)
Supplement: S1 Table — (DOCX) [file pone.0253722.s001.docx]

**S1 Table. Univariable analysis of PFS and OS**

|  |  | Overall Survival | | Progression Free Survival | |
| --- | --- | --- | --- | --- | --- |
|  |  | Median (95% CI) | P VALUE | Median (95% CI) | p value |
| Age(years) | <45 | 27.3 (27.3-NA) | 0.64 | 11.02 (3.09-NA) | 0.069 |
|  | 45-64 | 26.1 (16.62-NA) |  | 6.05 (4.96-9.23) |  |
|  | 65-74 | 23.1 (11.83-NA) |  | 11.96 (6.44-NA) |  |
|  | >75 | 21.9 (3.32-NA) |  | 23.62 (19.84-NA) |  |
| Menopausal status | Premenopausal | NA (16.8-NA) | 0.94 | 7.13 (3.98-12.5) | 0.061 |
|  | Postmenopausal | 27.1 (20.2-NA) |  | 8.05 (5.98-13.5) |  |
| Histology | Invasive ductal | 27.07 (21.88-NA) | 0.52 | 7.69 (5.98-12) | 0.9 |
|  | Invasive lobular | 8.44 (8.34-NA) |  | 6.24 (1.74-NA) |  |
| ECOG PS | PS1 | NA (NA-NA) | <0.001 | 15.21 (10.61-28.35) | 0.0016 |
|  | PS2 | 15.28 (11.83-27.1) |  | 6.05 (4.14-8.05) |  |
|  | PS >=3 | 8.34 (3.68-NA) |  | 3.88 (2.46-NA) |  |
| Line of therapy | First Line | NA (NA-NA) | <0.001 | 21.06 (16.36-NA) | <0.001 |
|  | Second Line | 27.3 (20.24-NA) |  | 8.18 (5.52-NA) |  |
|  | Third Line | 16.82 (11.83-NA) |  | 4.96 (2.89-15.2) |  |
|  | Fourth line | 27.07 (14.13-NA) |  | 6.28 (4.47-NA) |  |
|  | Fifth line | 8.44 (3.98-NA) |  | 4.3 (3.88-NA) |  |
|  | Others | 9.63 (7.75-NA) |  | 2.92 (1.31-NA) |  |
| HB | <=10.8 | 27.1 (16.6-NA) | 0.98 | 8.18 (6.31-15.2) | 0.16 |
|  | >10.8 | 26.1 (16.8-NA) |  | 5.39 (4.3-11) |  |
| Platelets | <=224 | 20.2 (12.9-NA) | 0.22 | 6.97 (4.96-11.4) | 0.59 |
|  | >224 | 27.3 (27.1-NA) |  | 9.53 (5.52-19) |  |
| TLC | <=5.2 | 27.1 (20.2-NA) | 0.9 | 7.13 (4.14-12) | 0.6 |
|  | >5.2 | 27.3 (16.6-NA) |  | 8.18 (5.98-19) |  |
| Absolute Neutrophils | <=2.6 | 38.5 (21.9-NA) | 0.2 | 6.31 (4.14-13.1) | 0.43 |
|  | >2.6 | 27.1 (12.9-NA) |  | 7.89 (5.98-13.5) |  |
| Total bilirubin | <=0.67 | 27.1 (16.8-NA) | 0.64 | 7.16 (5.98-12.5) | 0.56 |
|  | >0.67 | 27.3 (21.9-NA) |  | 7.89 (4.96-15.8) |  |
| Dose reductions | Yes | 20.2 (9-NA) | 0.23 | 8.02 (6.31-13.5) | 0.47 |
|  | No | 27.3 (26.1-NA) |  | 6.28 (4.96-13.1) |  |
| Dose reduction type | 125 - 100 mg | 20.2 (12.94-NA) | <0.001 | 9.23 (6.31-19.8) | <0.001 |
|  | 100-75 mg | NA (3.94-NA) |  | 10.61 (3.88-NA) |  |
|  | 125 - 75 mg | 2.2 (NA-NA) |  | 1.87 (NA-NA) |  |
| Partner drug | Letrozole | 27.3 (20.24-NA) | 0.005 | 16.36 (5.98-29.7) | 0.047 |
|  | Fulvestrant | 27.07 (16.62-NA) |  | 7.16 (5.52-12.6) |  |
|  | exemestane | NA (14.19-NA) |  | 3.96 (3.32-NA) |  |
|  | Letrozole+Leuprolide | NA (12.19-NA) |  | 7.89 (3.98-NA) |  |
|  | Fulvestrant+Leuprolide | 5.52 (2.04-NA) |  | 7.69 (1.05-NA) |  |
|  | Letrozole + others | 3.68 (NA-NA) |  | 2.69 (NA-NA) |  |
| ER Allred score | 3-6 | 11.8 (9.07-NA) | 0.097 | 4.96 (2.92-NA) | 0.97 |
|  | 7-8 | 27.1 (21.88-NA) |  | 7.89 (6.05-12) |  |
| PR Allred score | 3-6 | 26.1 (20.2-NA) | 0.96 | 7.69 (5.52-12) | 0.15 |
|  | 7-8 | 38.5 (9-NA) |  | 7.16 (4.96-28.4) |  |
| Previous line CT | First Line | 38.5 (NA-NA) | 0.075 | 5.98 (3.45-NA) | 0.014 |
|  | Second Line | NA (12.94-NA) |  | 11.01 (5.52-NA) |  |
|  | Third Line | 14.1 (8.44-NA) |  | 6.28 (3.88-8.02) |  |
|  | Fourth Line | 11 (3.02-NA) |  | 3.3 (1.31-NA) |  |
|  | Fifth line | NA (NA-NA) |  | 7.16 (NA-NA) |  |
| Previous line HT | First Line | 27.3 (16.62-NA) | 0.03 | 11.37 (5.52-NA) | 0.026 |
|  | Second Line | 27.1 (12.19-NA) |  | 7.89 (4.96-15.2) |  |
|  | Third Line | NA (9-NA) |  | 4.93 (3.09-13.1) |  |
|  | Fourth Line | 12.9 (3.32-NA) |  | 5.36 (1.38-NA) |  |
|  | Fifth line | NA (NA-NA) |  | 4.14 (NA-NA) |  |
| Bone vs. Visceral | Bone only | 38.5 (16.8-NA) | 0.8 | 21.06 (9.53-NA) | 0.044 |
|  | Visceral | 26.1 (20.2-NA) |  | 6.44 (5.39-10.6) |  |
| Line of therapy | First line | NA (NA-NA) | <0.001 | 21.06 (16.36-NA) | <0.001 |
|  | Second line | 27.3 (20.2-NA) |  | 8.18 (5.52-NA) |  |
|  | Third line or later | 14.2 (11.8-NA) |  | 5.36 (4.14-7.16) |  |
| Prior CT | No | 27.3 (26.1-NA) | 0.022 | 16.36 (12-25.26) | 0.014 |
|  | Yes | 23.1 (14.1-NA) |  | 5.98 (4.4-7.89) |  |
| Prior HT | No | NA (21.9-NA) | 0.13 | 19.84 (12.52-NA) | 0.098 |
|  | Yes | 27.1 (16.8-NA) |  | 6.97 (5.39-9.53) |  |

Abbreviations used: CT, chemotherapy; HT, hormone therapy; PS, performance status; ER, estrogen receptor; PR, progesterone receptor ; TLC, total leucocyte count
